# Simulation Analysis of Thermal Deformation and Extruded Profile Formability of Al–10Mg–3Zn Aluminum Alloy

**DOI:** 10.3390/ma19020375

**Published:** 2026-01-17

**Authors:** Guanmei Niu, Wei Li, Kaidi Jiang, Yang Yang, Guojun Wang, Cheng Liu, Linzhong Zhuang

**Affiliations:** 1 State Key Laboratory for Advanced Metals and Materials, University of Science and Technology Beijing, Beijing 100083, China; niu_gm133@chinalco.com.cn; 2 Beijing Advanced Innovation Center for Materials Genome Engineering, University of Science and Technology Beijing, Beijing 100083, China; 3 Chinalco Material Application Research Institute Co., Ltd., Beijing 102209, China

**Keywords:** Al–10Mg–3Zn alloy, flow stress, hot processing maps, response surface method

## Abstract

To investigate the hot deformation characteristics of the Al–10Mg–3Zn alloy, a series of hot compression tests was carried out using a Gleeble-3500 simulator. The experimental matrix covered temperatures of 300–450 °C and strain rates from 0.001 to 10 s^−1^. The true stress–strain curves were obtained and the hot processing map of the alloy was constructed based on the Dynamic Material Model principle. The multi-objective optimization of the extrusion process parameters was performed using the response surface method. The results showed that the flow stress of Al–10Mg–3Zn alloy increased with the increase in the strain rate and decreased with the increase in the deformation temperature, indicating that the alloy had a positive strain rate sensitivity. A strain-compensated Arrhenius constitutive model and a hot processing map of Al–10Mg–3Zn alloy were established based on the temperature-corrected data; here, the optimal temperature range and strain rate range for hot processing were specified. The optimal extrusion process parameters, determined by the response surface method, were as follows: billet temperature of 400 °C, extrusion speed of 0.20 mm/s, and ingot length of 350 mm. With this parameter combination, the simulation predicted an extrusion load of 73.29 MN, a velocity deviation of 24.96%, and a cross-sectional temperature difference of 9.48 °C for the profile. The predicted values from the response surface method were highly consistent with those from the finite element simulation. The optimized process parameters significantly reduced the extrusion load of the profile.

## 1. Introduction

Al–Mg alloys have become pivotal structural materials in the modern transportation industry, including marine, aerospace, and automotive manufacturing; this is due to their exceptional specific strength, excellent toughness, and outstanding corrosion resistance [1,2]. However, with the growing demand for lightweight transportation vehicles, the mechanical properties of traditional Al–Mg alloys (such as those in the 5XXX series) are no longer sufficient to meet the stringent requirements for tough, high-strength materials in next-generation equipment manufacture [3,4,5,6]. In the domain of engineering applications, high-magnesium–aluminum alloy extrusions have exhibited considerable potential in the context of rail transportation. In order to address the pressing demand for lightweight materials in next-generation high-speed trains, there is an urgent need to develop new aluminum alloys that combine high strength (target tensile strength ≥ 500 MPa), low density (target density ≤ 2.7 g/cm^3^), and excellent corrosion resistance and weldability.

As a non-heat-treatable strengthened aluminum alloy, the strength of Al–Mg alloys primarily relies on solid solution strengthening and work hardening. Increasing the Mg content in the alloy is one of the most direct and effective methods for enhancing the strength of Al–Mg alloys [7]. However, when the Mg content exceeds 3.5 wt.%, the β-Al3Mg phase tends to continuously precipitate at grain boundaries. Due to its lower electrochemical potential (−1.15 V) compared to that of the aluminum matrix (−0.82 V), the β phase acts as an anode and preferentially dissolves in corrosive media, reducing the alloy’s resistance to intergranular corrosion and limiting the application of high-Mg Al–Mg alloys [8,9]. Precipitation strengthening is one of the most effective strengthening mechanisms in aluminum alloys. In recent years, researchers have found that adding a small amount (less than 1.0 wt.%) of Zn to traditional Al–Mg alloys improves their corrosion resistance [10,11,12,13,14,15]. Simultaneously, after stabilization treatment, the addition of a small amount of Zn can enhance the yield strength of Al–Mg alloys, which is attributed to the synergistic effects of solid solution strengthening and precipitation strengthening [11,15]. In a study on the hot deformation behavior of Al–Zn–Mg–Cu–Zr series alloys, Maxim G. Khomutov et al. [16] systematically investigated the influence of varying Sc contents (0.05%, 0.1%, and 0.15%) on the deformation behavior of the alloys under conditions of 300–450 °C and 0.1–15 s^−1^ through hot compression experiments. The analysis indicated that the optimal hot processing window for alloys containing 0.05% and 0.1% Sc is 350–400 °C and 0.1–1 s^−1^; meanwhile, for the alloy with 0.15% Sc, the optimal strain rate within the same temperature range narrows to 0.1 s^−1^. This optimized processing domain corresponds to a peak power dissipation efficiency of approximately 30–35% and lies within the flow stability region. For an as-cast Al–5Mg–3Zn–1Cu alloy, Lei et al. [17] determined its optimal hot processing parameter window by constructing processing maps and indicated that dynamic recrystallization is the dominant softening mechanism under high-temperature and low-strain-rate conditions; they identified that its flow instability behavior is also highly sensitive to temperature and strain rate. For high-alloyed Al–Mg–Zn alloys containing multiple insoluble phases, the selection of hot deformation parameters requires a critical balance. Yang et al. [18] demonstrated that, for a cast Al–9.7Mg–2.9Zn alloy, rich in Mn and Cr, high strain rates (e.g., 10 s^−1^) significantly promote dynamic recrystallization; however, this comes at the cost of severe cracking, induced by coarse AlMgMnTiCr particles. Their findings suggest that, for such alloys, a lower strain rate is essential to mitigate cracking; a compositional adjustment can also reduce the presence of crack-inducing elements (Mn, Cr), and this is recommended for improved workability.

High-Mg–aluminum alloys face significant challenges during the extrusion forming process. These include extremely high deformation resistance, which can lead to defects such as ‘stalling’ (extruder overload shutdown), ‘peeling’ (surface cracking), and ‘waviness’ (dimensional fluctuations). In severe cases, extrusion cracking may occur. Currently, systematic research on the extrusion process of Al–Mg alloys (particularly those with high Mg content) remains relatively scarce. Due to the narrow thermal processing window of high-Mg–aluminum alloys, the traditional trial-and-error process development not only takes a long time but also incurs high costs. To address this challenge, the present study employs an advanced approach, combining material characterization with numerical simulation: first, through thermal compression testing (temperature range: 300–450 °C; strain rate range: 0.001–10 s^−1^), we systematically investigated the high-temperature rheological behavior of an Al–10Mg–3Zn alloy and established an Arrhenius constitutive model based on strain compensation; secondly, based on the Dynamic Material Model (DMM) theory, we constructed a thermal processing map for the alloy, clearly defining the safe processing zones and instability zones under different strain conditions; finally, using professional finite element (FE) software Qform UK11.0, an FE model of the extrusion process for railway vehicle profiles was established to thoroughly analyze the influence of the extrusion process parameters on forming quality (including temperature field distribution, extrusion load evolution, and metal flow velocity uniformity), thereby determining the optimal process parameter window. This study not only provides theoretical guidance and technical support for the industrial production of high-magnesium–aluminum alloy extrusions but also significantly promotes the application of such advanced materials in high-end equipment manufacturing sectors, such as high-speed train manufacturing.

## 2. Materials and Simulation Methods

### 2.1. Materials and Experimental Methods

Al–10Mg–3Zn alloy ingots were prepared in the laboratory, and the chemical composition of the as-cast alloy was analyzed using a direct-reading spectrometer (Germany SPECTRO LAVM11 Direct-Reading Spectrometer). The measurement results are shown in Table 1.

Al–10Mg–3Zn alloy ingots were subjected to a homogenization treatment at 430 °C for a duration of 24 h. A set of ∅10 mm × 15 mm hot compression cylindrical specimens were cut at the midpoint of the radius. They were tested on a Gleeble-3500 thermal simulation testing machine(Dynamic Systems Inc. (DSI) in the United States) at temperatures of 300 °C, 330 °C, 360 °C, 390 °C, 420 °C, and 450 °C with strain rates of 0.001 s^−1^, 0.01 s^−1^, 0.1 s^−1^, 1 s^−1^, and 10 s^−1^. Each sample was heated at a rate of 10 °C per minute to the target temperature, held at that temperature for 60 s, and then subjected to thermal compression. The samples were compressed to a total deformation of 60% and then immediately water-quenched.

**Figure 1:**
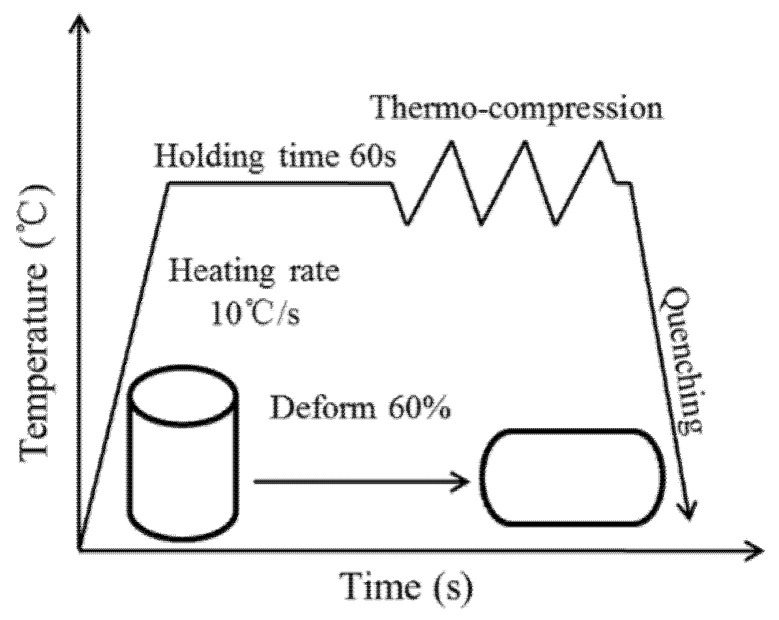
Thermal compression experiment flowchart.

**Figure 2:**
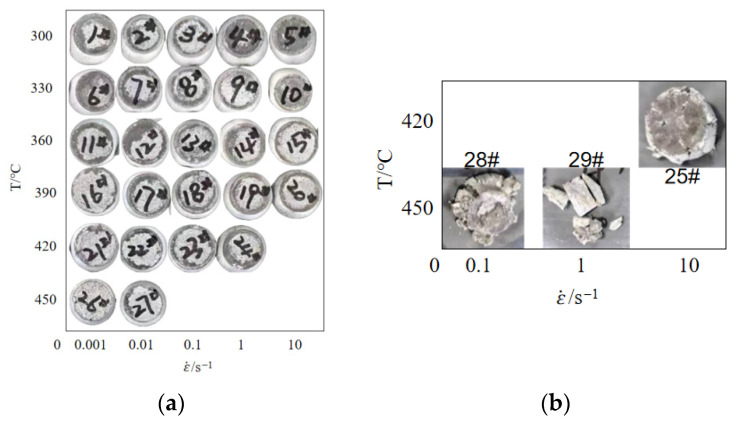
Test specimens after heat compression deformation: (**a**) normal sample; (**b**) broken sample.

The test process is presented in Figure 1, while the samples following thermal compression deformation are shown in Figure 2. Figure 2 shows that the hot-compressed specimens exhibit a certain degree of bulging. Although graphite lubricant was used during the hot-compression process to reduce the influence of friction on the test results, it was not possible to completely eliminate friction. Based on a simplified estimation derived from the geometry of the bulged sample after compression, the estimated error in flow stress due to friction is within ±5%. This falls within the typical scatter band of such tests and does not alter the fundamental trends regarding the alloy’s sensitivity to temperature and strain rate. The model established in this study is intended for direct input into macroscopic finite element simulations of the extrusion process, where the influence of friction correction on the simulation results remains within an acceptable range. The influence of friction will be considered in future precise studies on material deformation.

### 2.2. Model Establishment and Simulation Plan

**Figure 3:**
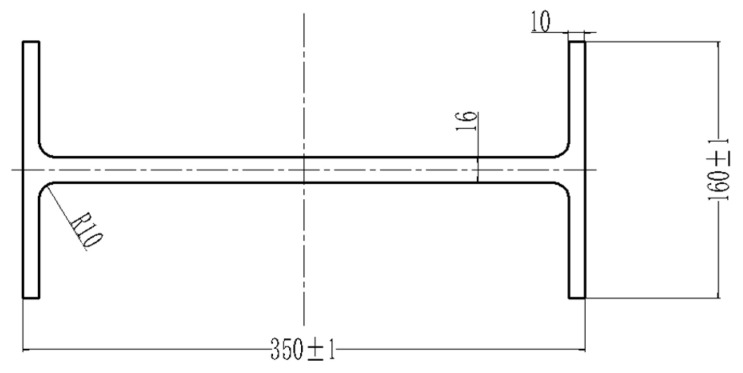
Cross-section diagram of extruded profiles for rail transit.

**Figure 4:**
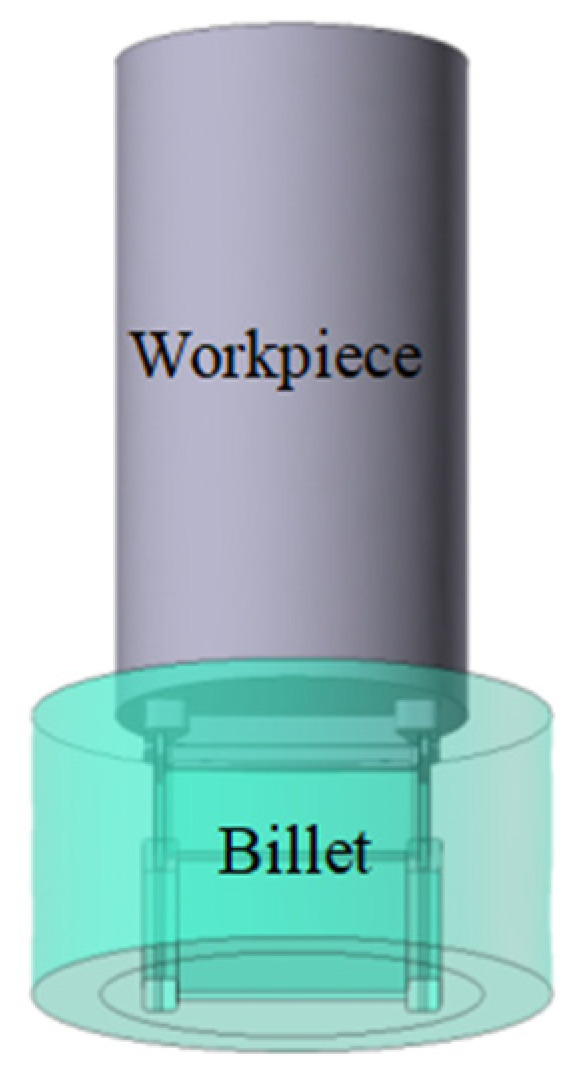
FE model of the extrusion process for rail transit profiles.

In this study, a steady-state extrusion simulation model for rail transit Al–10Mg–3Zn aluminum alloy profiles was established using the Qform finite element software. The simulation employed a Lagrangian–Eulerian method for calculation. The model featured an initial billet of Ø450 mm × 800 mm; the mesh comprised roughly 4.5 million tetrahedral elements. The boundary conditions were set as follows: a shear friction model was applied with a friction factor of 1, and the heat transfer coefficient was set to 30,000 W/(m^2^·K), primarily accounting for thermal exchange between the billet and the die, the extrusion container, and the extrusion pad. The billet was heated to 390 °C and extruded at a speed of 0.3 mm/s. Figure 3 presents a cross-sectional view of the profile, while Figure 4 shows the FE model of the extrusion process. A simulation system was utilized to analyze the effects of the billet length, the billet temperature, the extrusion speed, and the friction coefficient on the extrusion formability of the extruded profiles. Detailed simulation schemes are presented in Table 2 and Table 3. The effects of the extrusion temperature, extrusion speed, and overall friction coefficient on the properties of the extruded profiles (simulation schemes I–VII) are investigated in Table 2. The effects of billet length and extrusion cylinder friction coefficient on the properties of the extruded profiles (simulation schemes A–E) are investigated in Table 3.

## 3. Results and Discussion

### 3.1. Stress–Strain Curve and Temperature Correction

**Figure 5:**
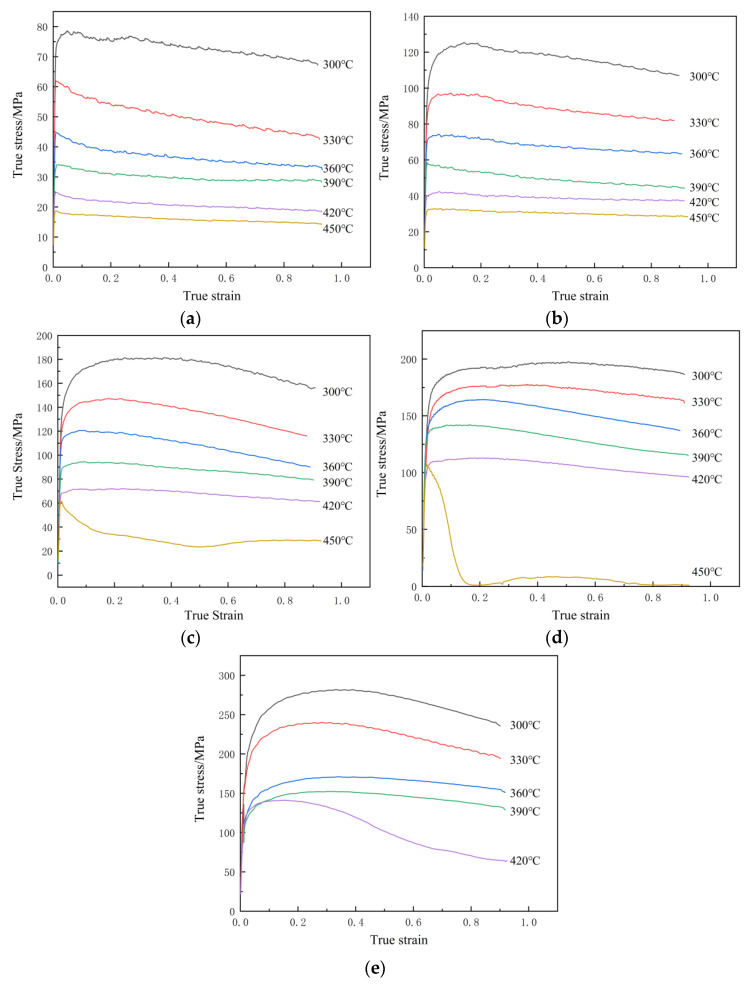
True stress–true strain curves of Al–10Mg–3Zn alloy under different deformation conditions. Each figure refers to a different strain rate: (**a**) 0.001 s^−1^; (**b**) 0.01 s^−1^; (**c**) 0.1 s^−1^; (**d**) 1 s^−1^; (**e**) 10 s^−1^.

Figure 5 presents the true stress–strain curves of the Al–10Mg–3Zn alloy under various hot compression conditions. The curves exhibit a transient stage followed by a steady-state stage [19,20]. Initially, rapid work hardening dominates due to dislocation multiplication. As strain accumulates, the stored deformation energy drives dynamic recovery and recrystallization, which gradually counteract the hardening. Ultimately, a steady-state flow stress is reached when a balance is achieved between these competing mechanisms [21]. Consistently, flow stress decreases with increasing temperature at a fixed strain rate, and increases with strain rate at a constant temperature due to more pronounced work hardening [22].

As illustrated in Figure 5, from the complete dataset, three instances of anomalous behavior were observed in the true stress–strain curves. For the case of a temperature of 420 °C and strain rate of 10 s^−1^ (purple curve in Figure 5e), the stress exhibited a linear decrease after the strain exceeded 0.2. As illustrated in Figure 2b, this sample, 25, exhibited signs of cracking during the compression process. Furthermore, cracking was also observed in samples subjected to deformation at a temperature of 450 °C and a strain rate greater than 0.1 s^−1^ (samples 28 and 29 in Figure 2b). This finding suggests that thermal plastic instability occurred at elevated temperatures and high strain rates, with dislocation proliferation rates exceeding dynamic recovery rates; this ultimately led to a dislocation pile-up and micro-pore formation. Coupled with insufficient thermal activation energy, this ultimately resulted in intergranular or transgranular cracking [23]. Therefore, the true stress–strain curve data under these conditions are unreliable and are excluded from the subsequent constitutive modeling.

The temperature rise in the sample during hot compression is primarily due to deformation and friction heating; this, coupled with the insufficient responsiveness of the temperature control device (which fails to compensate in real time), leads to a deviation between the actual and preset temperatures.

**Figure 6:**
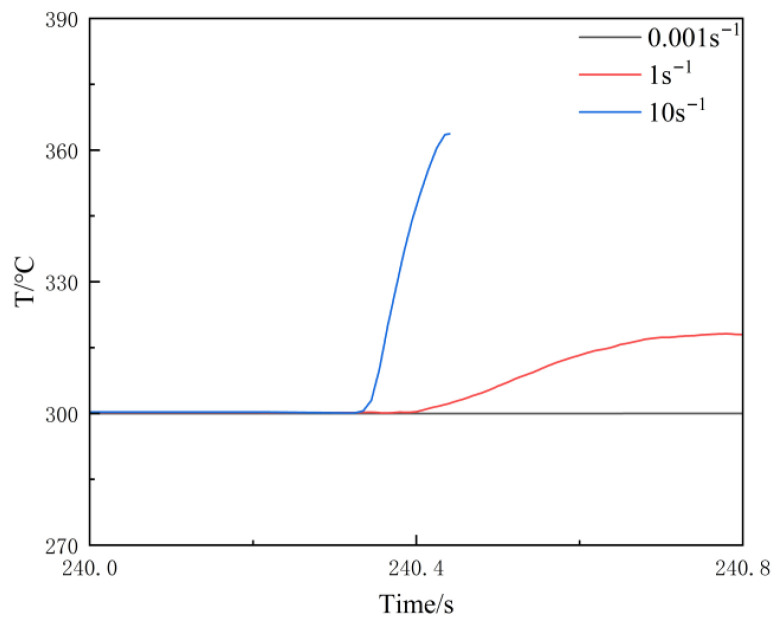
Temperature rise curves during compression processes at identical temperatures but with varying strain rates.

Figure 6 shows that, at 300 °C, temperature rises of approximately 63 °C, 17 °C, and a negligible increase were observed during compression at strain rates of 10 s^−1^ (blue curve), 1 s^−1^ (red curve), and 0.001 s^−1^ (black curve), respectively. The test results obtained at high strain rates cannot accurately reflect the actual plastic deformation process of the specimen. Therefore, it is necessary to temperature-correct the collected data [24,25,26,27,28]. The overburning point of the homogenized Al-10Mg-3Zn high-magnesium low-density alloy was determined to be 735 K through testing (for details, see Figure 13). A correction was then applied based on the modification method described in [27].

**Figure 7:**
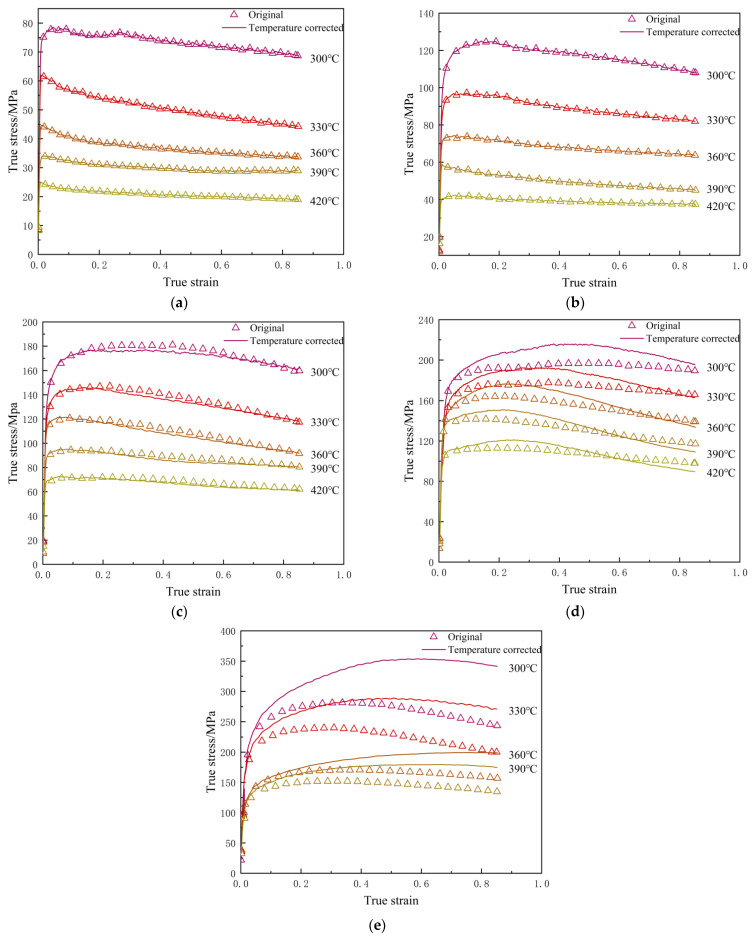
Comparison of the measured true stress–true strain curve with the temperature-corrected curve. Each figure refers to a different strain rate: (**a**) 0.001 s^−1^; (**b**) 0.01 s^−1^; (**c**) 0.1 s^−1^; (**d**) 1 s^−1^; (**e**) 10 s^−1^.

Figure 7 shows a comparison of the true stress–strain curves before and after temperature correction. It can be observed that, during compression at low strain rates (<1 s^−1^) and different temperatures, almost no temperature rise occurred, and the true stress–strain curves before and after correction are essentially consistent. In contrast, during high-strain-rate (≥1 s^−1^) compression, a significant temperature rise was observed, with more pronounced effects at lower temperatures and higher strain rates. The difference in flow stress before and after correction was most significant under these conditions. Specifically, during compression at 300 °C and under a strain rate of 10 s^−1^, the temperature increased by approximately 63 °C; this led to a maximum difference in true stress of up to 100 MPa at the same strain level before and after correction. The measured flow stress is lower than that of its corrected counterpart. This can be attributed to the substantial adiabatic heating induced during high-strain-rate deformation. The elevated temperature softens the material, improving its plasticity and decreasing its flow resistance, which consequently depresses the measured stress value.

### 3.2. Constitutive Equation

Using the temperature-corrected true stress–strain data, the Arrhenius constitutive equation was employed to establish the relationship between the flow stress and the key deformation parameters (temperature, strain rate, and strain) for the alloy. The effects of the temperature and strain rate are represented by the Zener–Hollomon parameter, *Z*. The dependence of flow stress on the *Z*, expressed by the hyperbolic sine law in the Arrhenius constitutive model [29,30], is given as follows:

(1) $$Z=\dot{\varepsilon }\exp(Q/RT)$$

(2) $$\dot{\varepsilon }=AF\left(\sigma \right)\exp\left[-Q/\left(RT\right)\right]$$

Among these terms, F(σ) is a function of stress:

(3) $$F\left(\sigma \right)=\left\{\begin{array}{c} \sigma^{n_{1}}(\alpha \sigma <0.8)\\ exp\left(\beta \sigma \right)(\alpha \sigma >1.2)\\ {\left[\begin{array}{c} sinh\left(\alpha \sigma \right) \end{array}\right]}^{n}\left(\mathrm{general}\;\mathrm{validity}\right) \end{array}\right.$$

In the above equation, the hyperbolic sine function, F(σ), describes the thermal rheological stress, where σ is the flow stress (MPa), $\dot{\varepsilon }$ is the strain rate (s^−1^), T is the temperature (K), and Q is the activation energy for thermal deformation $\left(J\cdot mol^{-1}\right)$. The parameters R (the ideal gas constant, $J\cdot mol^{-1}\cdot K^{-1}$), A, α, β, and $n_{1}$ are material constants, with the relationship $\alpha =\beta /n_{1}$ holding among them.

(4) $$\dot{\varepsilon }=A{\left[\begin{array}{c} sinh\left(\alpha \sigma \right) \end{array}\right]}^{n}\exp\left[-Q/\left(RT\right)\right]$$

By combining Equations (1) and (4), the Zener–Hollomon parameter, Z, is defined as follows:

(5) $$Z=A{\left[sinh\left(\alpha \sigma \right)\right]}^{n}$$

According to the definition of the hyperbolic sine function, the flow stress can be expressed in terms of the Zener–Hollomon parameter, Z.

(6) $$\sigma =\frac{1}{\alpha }ln\left\{{\left(\frac{Z}{A}\right)}^{\frac{1}{n}}+{\left[{\left(\frac{Z}{A}\right)}^{\frac{2}{n}}+1\right]}^{\frac{1}{2}}\right\}$$

Next, we take the case in which the strain value is 0.2 as an example to introduce the determination process of material constant solutions under the entire range of stress conditions. Substitute Equation (3) into Equation (4), and take the logarithm of both sides. Then, Equation (7) can be obtained:

(7) $$\left\{\begin{array}{c} ln\left(\sigma \right)=\frac{1}{n_{1}}ln\left(\dot{\varepsilon }\right)-\frac{1}{n_{1}}ln\left(B\right)\\ \sigma =\frac{1}{\beta }ln\left(\dot{\varepsilon }\right)-\frac{1}{\beta }ln\left(C\right)\\ ln\left[sinh\left(\alpha \sigma \right)\right]=\frac{Q}{nR}\frac{1}{T}+\frac{\left(\ln\dot{\varepsilon }-\mathrm{lnA}\right)}{n} \end{array}\right.$$

**Figure 8:**
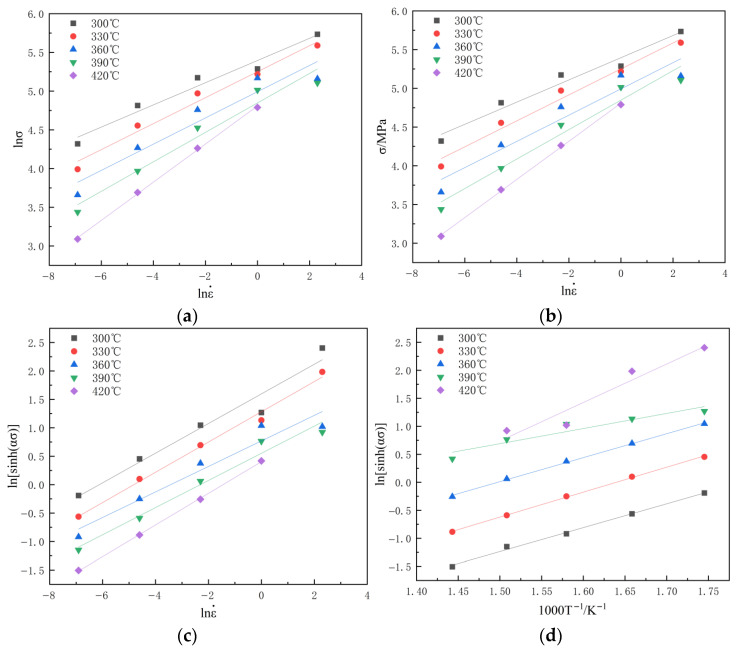
Homogeneous Al–10Mg–3Zn alloy relationship curves: (**a**) $\ln\sigma -\ln\dot{\varepsilon }$; (**b**) $\sigma -\ln\dot{\varepsilon }$; (**c**) $\ln[\sin h(\alpha \sigma )]-\ln\dot{\varepsilon }$; (**d**) ln[sinh(ασ)]−1/T.

The stress values at a strain of 0.2 were obtained experimentally and extracted from Figure 7 for coefficient calibration. Subsequently, the linear fitting of these data (Figure 8a,b) yielded the parameters $n_{1}=5.631$ and $\beta =0.056\;{MPa}^{-1}$, from which $\alpha =\beta /n_{1}=0.010\;{MPa}^{-1}$ was derived. The parameter n was then determined to be 3.967 from the slope of the ln[sinh(ασ)] vs. $\ln\dot{\varepsilon }$ plot (Figure 8c). Finally, from the ln[sinh(ασ)] vs. 1/T relationship (Figure 8d), the activation energy, *Q*, and the material constant, *A*, were calculated as 148,900 J/mol and 6.028 × 10^10^, respectively. Thus, the constitutive model based on the hyperbolic sine function for ε=0.2 can be established as follows:

(8) $$\dot{\varepsilon }=6.038\times 10^{10}{\left[sinh\left(0.0100\sigma \right)\right]}^{3.967}exp\left[-148900/\left(RT\right)\right]\;$$

The formula does not explicitly consider the effect of strain. However, the deformation activation energy, *Q*, and the material constants α, n, and ln*A* have been shown to vary with strain [30]. It is assumed that their values are all polynomial functions of the strain *ε*. To determine this function, parameter values obtained at different strain levels ranging from 0.05 to 0.80 with an interval of 0.05 were used to fit the polynomial function. To ensure greater accuracy in the fitting results, a fifth-order polynomial function was employed to fit the relevant material parameters.

Ultimately, the strain-compensated Arrhenius constitutive model for the Al–10Mg–3Zn alloy was formulated as Equation (9). With the material parameters determined, this model enables the prediction of flow stress for a wide range of deformation conditions.

(9) $$\sigma =\frac{1}{\alpha (\varepsilon )}\ln\{{\left(\frac{\dot{\varepsilon }\exp[Q(\varepsilon )/8.314T]}{A(\varepsilon )}\right)}^{\frac{1}{n(\varepsilon )}}+{\left[{\left(\frac{\dot{\varepsilon }\exp[Q(\varepsilon )/8.314T]}{A(\varepsilon )}\right)}^{\frac{2}{n(\varepsilon )}}+1\right]}^{\frac{1}{2}}\}$$

**Figure 9:**
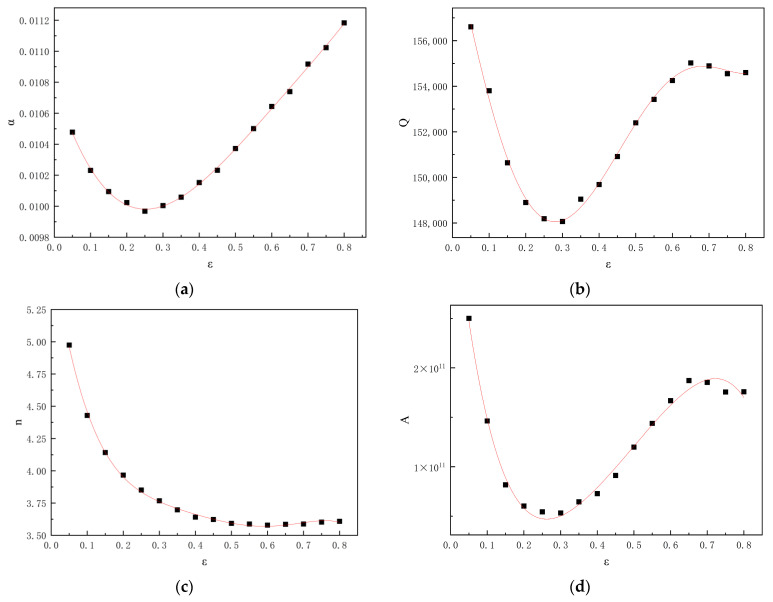
Parameter variation under different strains: (**a**) α-ε; (**b**) Q-ε; (**c**) n-ε; (**d**) A-ε.

In the equation above, R is the gas constant (8.314 $J\cdot mol^{-1}\cdot K^{-1}$), and the terms *α*(*ε*), *Q*(*ε*), ln*A*(*ε*), and *n*(*ε*) are polynomial functions describing the dependence of α, *Q*, ln*A*, and *n* on strain, respectively.The correlation coefficients in the fitted polynomial function (10) were determined by fitting the variation curves of each parameter with strain shown in Figure 9. The coefficients of each calibrated polynomial function are shown in Table 4. Substituting these correlation coefficients yields the calibrated polynomial functions for each parameter, as shown in Formula (10):

(10) $$\begin{array}{l} \alpha (\varepsilon )=0.01079-0.00734\varepsilon +0.01996\varepsilon^{2}-0.01405\varepsilon^{3}-0.0022\varepsilon^{4}+0.00482\varepsilon^{5}\\ Q(\varepsilon )=160458-75667\varepsilon -7381\varepsilon^{2}+737554\varepsilon^{3}-1291052\varepsilon^{4}+642526\varepsilon^{5}\\ n(\varepsilon )=5.74-18.09\varepsilon +76.1\varepsilon^{2}-160.36\varepsilon^{3}+167.69\varepsilon^{4}-67.91\varepsilon^{5}\\ A(\varepsilon )=3.99\times 10^{11}-3.69\times 10^{12}\varepsilon +1.4\times 10^{13}\varepsilon^{2}-2.49\times 10^{13}\varepsilon^{3}+2.36\times 10^{13}\varepsilon^{4}-9.7\times 10^{12}\varepsilon^{5} \end{array}$$

### 3.3. Hot Processing Map

The Dynamic Material Model (DMM) [31,32,33,34] postulates that, during thermal deformation, metals constitute a closed energy dissipation system. The external power input, *P*, at a given strain rate is accordingly separated into the dissipative component, *G*, and the co-dissipative component, *J*, which is expressed mathematically in Equation (11):

(11) $$P=\sigma \dot{\varepsilon }=G+J=\int_{0}^{\dot{\varepsilon }}\sigma d\dot{\varepsilon }+\int_{0}^{\sigma }\dot{\varepsilon }d\sigma$$

where *G* represents the energy dissipated through material deformation (dissipation), and *J* denotes the energy consumed by microstructural evolution (co-dissipation). The strain rate sensitivity index, *m* (Equation (12)), quantitatively reflects the competition between these two processes.

(12) $$m=\frac{dJ}{dG}=\frac{\dot{\varepsilon }d\sigma }{\sigma d\dot{\varepsilon }}=\frac{dln\sigma }{dln\dot{\varepsilon }}$$

To quantify the efficiency of power dissipation during the pronounced microstructural evolution, the parameter *η* is introduced. It attains its specific form under the condition of maximum co-dissipation, i.e., when *m* = 1 and *J = J_max_*, as follows:

(13) $$\eta =\frac{J}{J_{max}}=\frac{2m}{m+1}$$

The hot processing properties of materials need to be judged in combination with instability maps, which are developed based on the principle of irreversible thermodynamics of fluids. The basis for determining instability is shown in Equation (14):

(14) $$\xi (\dot{\varepsilon })=\frac{\partial \ln(\frac{m}{m+1})}{\partial \ln\dot{\varepsilon }}+m<0$$

In the equation, $\xi (\dot{\varepsilon })$ is the instability parameter. When $\xi \left(\dot{\varepsilon }\right)$ < 0, the material undergoes thermal deformation in an unstable state; defects will form in the internal structure.

Using Equations (13) and (14), the parameters η and $\xi (\dot{\varepsilon })$ were calculated across different deformation conditions. Two-dimensional contour maps—namely, the power dissipation map (η) and the flow instability map ($\xi (\dot{\varepsilon })$)—were then plotted as functions of strain rate and temperature. Superimposing the flow instability map onto the power dissipation map yields comprehensive hot processing maps.

**Figure 10:**
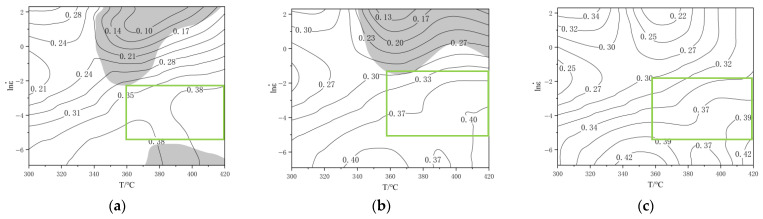
Homogeneous Al–10Mg–3Zn alloy hot processing map: (**a**) ε: 0.2; (**b**) ε: 0.4; (**c**) ε: 0.6.

Figure 10 presents the hot processing maps of the Al–10Mg–3Zn alloy at varying strains. The black line represents the power dissipation contour, the gray shaded area indicates the deformation instability zone, and the green box marks the deformation-safe region. At *ε* = 0.2, unstable regions (dark grey) appear under two conditions: (i) strain rates > 0.121 s^−1^ with *T* ≥ 342 °C; (ii) strain rates between 0.001 and 0.004 s^−1^ with *T* ≥ 373 °C. Both of these conditions exhibit strain-rate-sensitive temperature boundaries. As the strain increases to 0.4, the instability zone shifts and shrinks, concentrating at strain rates > 0.271 s^−1^ and *T* > 341 °C; notably, deformation cracking occurred at 420 °C and 10 s^−1^, consistent with the map’s prediction. By *ε* = 0.6, the instability zone completely disappears.

Therefore, the identified safe processing parameters for the Al–10Mg–3Zn alloy are 360–420 °C and 0.004–0.121 s^−1^, which corresponds to the green rectangular area shown across Figure 10a–c.

### 3.4. Extrusion Simulation Results

#### 3.4.1. Analysis of Simulation Results

**Figure 11:**
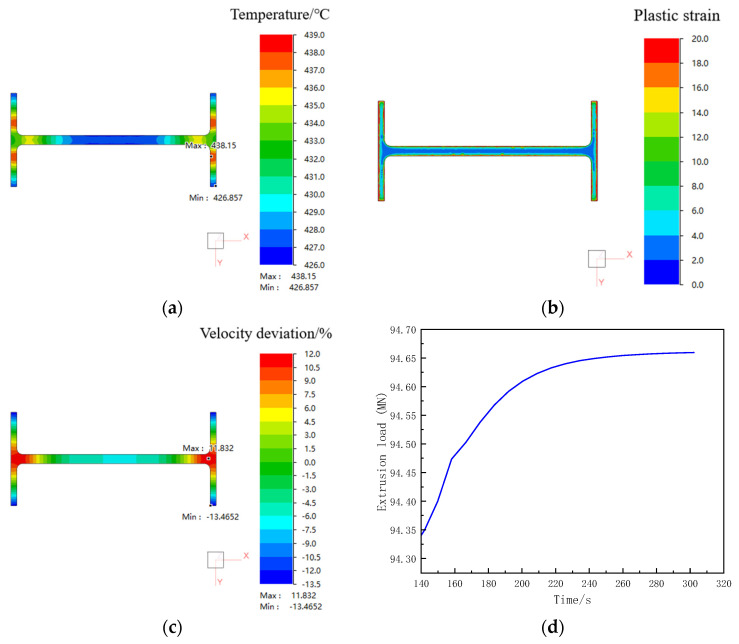
Prediction of profile formability under initial process conditions: (**a**) temperature field; (**b**) plastic strain field; (**c**) velocity deviation; (**d**) extrusion load.

Figure 11 shows the prediction of profile formability under the initial process conditions. As shown in the figure, the temperature variation across the profile cross-section is about 11 °C, spanning from a minimum of 427 °C to a maximum of 438 °C. The plastic strain distribution across the profile cross-section is non-uniform, with a surface layer exhibiting a high-strain zone of a certain depth. The maximum strain reaches 91, while the calculated average strain value is 8.36. The velocity deviation across the extruded profile cross-section is approximately 25%, indicating poor flow uniformity. The breakthrough extrusion load for the profile reaches 94 MN; this means that equipment with a capacity of at least 100 MN is required for successful extrusion.

#### 3.4.2. The Effect of Process Parameters on the Forming Temperature of Extruded Profiles

This section will explore the temperature evolution of the Al–10Mg–3Zn alloy during extrusion and its impact on profile quality, grounded in the established thermodynamic and heat transfer theories. Extrusion speed directly influences temperature distribution by affecting the generation rate of deformation heat, while the billet’s initial temperature determines the system’s thermal baseline. Differential Scanning Calorimetry (DSC) curves are employed to evaluate material thermal stability, particularly overburning temperature, which is a critical parameter for determining the processing limits of extrusion techniques.

**Figure 12:**
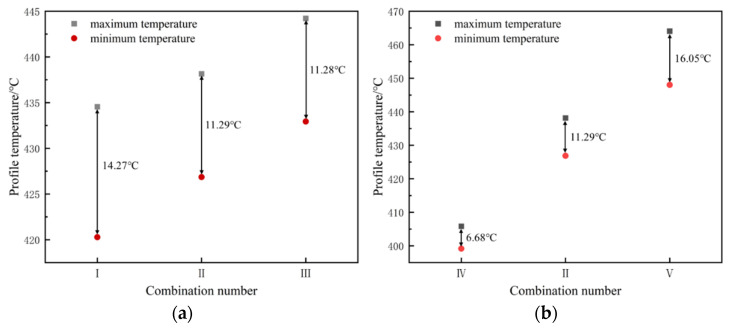
Effect of process parameters on the temperature field at the extrusion die exit: (**a**) billet temperature; (**b**) extrusion speed. In both considered cases, the parameter under investigation in a given figure displays a rise from left to right, see Table 2.

**Figure 13:**
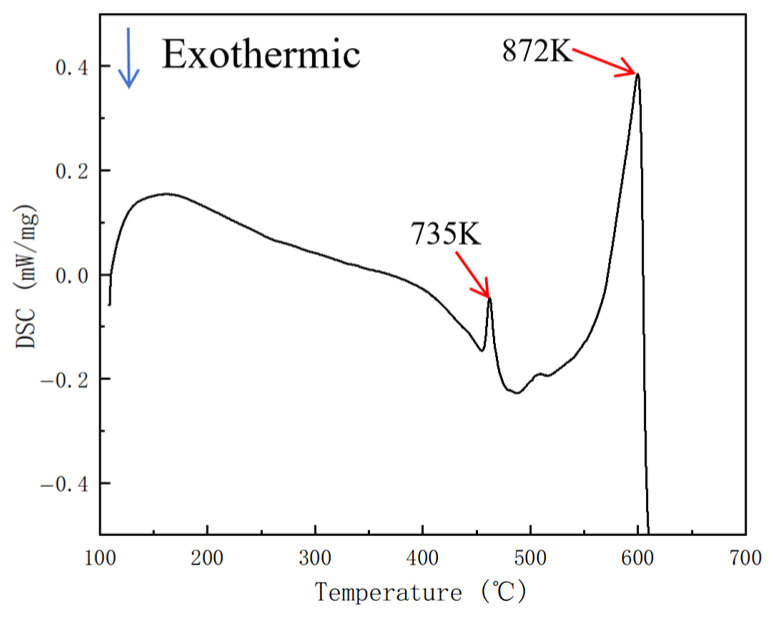
DSC curves of homogeneous Al–10Mg–3Zn alloy.

Figure 12 shows that increasing both the billet temperature and the extrusion speed will, to some extent, increase the overall extrusion temperature of the profile; Figure 12a indicates that increasing the billet temperature reduces the temperature difference within the profile cross-section, but the reduction is relatively small; Figure 12b shows that increasing the extrusion speed significantly increases the temperature difference within the profile cross-section. In simulation scheme V (extrusion speed 0.5 mm/s), the temperature difference in the profile cross-section is the largest, at approximately 16 °C. The extrusion speed has a greater influence on the extrusion temperature value and uniformity of the profile than the billet temperature. Figure 13 shows the DSC curves of a homogeneous Al–10Mg–3Zn alloy. As shown in Figure 13, the first endothermic peak corresponds to the incipient melting (overburning) temperature of the homogenized Al–10Mg–3Zn alloy, with a value of 462 °C. The maximum temperature of the extruded profile in scheme V is approximately 465 °C, which exceeds the overburning temperature. Therefore, the extrusion speed of 0.5 mm/s has reached the upper limit value.

#### 3.4.3. The Effect of Process Parameters on Extrusion Load

**Figure 14:**
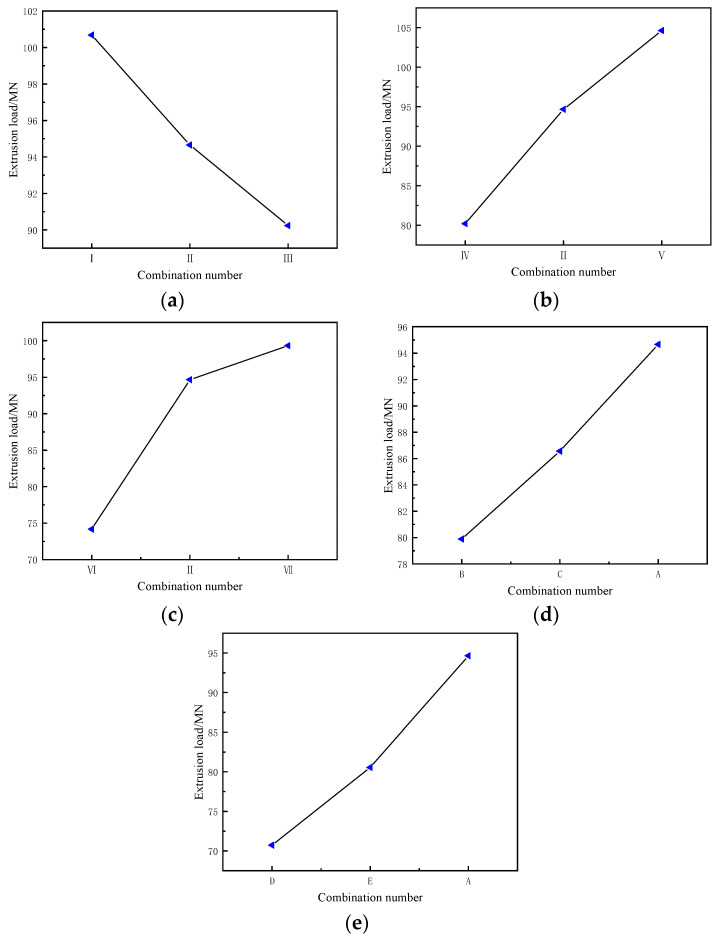
The effect of process parameters on the extrusion load of extruded profiles: (**a**) temperature; (**b**) extrusion speed; (**c**) overall friction coefficient; (**d**) ingot length; (**e**) extrusion cylinder friction coefficient. In all cases considered, the parameter under investigation in a given figure displays a rise from left to right; see Table 2 and Table 3.

Figure 14 shows the influence of different process parameters on the variation in extrusion load during profile extrusion. When the ingot length, extrusion speed, and friction coefficient increase, the extrusion load generally exhibits an upward trend (Figure 14b–e). This is because, with the growth of these parameters, the energy required to overcome the force of friction and material deformation resistance during the extrusion process escalates correspondingly, driving the extrusion load of the profile to rise. Conversely, when the billet temperature rises, the material’s deformation resistance diminishes (Figure 14a). This enhancement in the uniformity of metal flow and deformation during extrusion reduces the additional stresses induced by unevenly coordinated deformation, ultimately causing the extrusion load to decrease as the billet temperature increases.

#### 3.4.4. The Effect of Process Parameters on the Uniformity of Extrusion Flow Rate for Extruded Profiles

This paper adopts the velocity deviation difference (VDD) as an indicator to evaluate the uniformity of the profile’s exit speed. The velocity deviation difference is defined as follows:

$$VDD=(\frac{\nu_{max}-\nu_{min}}{\bar{\nu }})\%$$

In the formula, $\nu_{max}$ represents the maximum flow velocity of the nodes on the cross-section of the profile; $\nu_{min}$ represents the minimum flow velocity of the nodes on the cross-section of the profile; $\bar{\nu }$ represents the average velocity of all nodes on the cross-section of the profile. The smaller the velocity deviation difference (VDD), the more uniform the velocity distribution on the cross-section of the profile.

**Figure 15:**
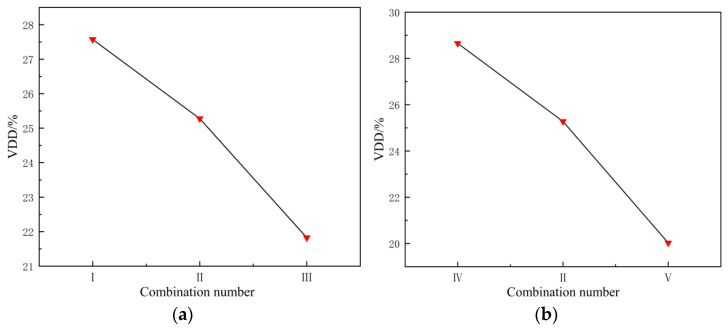
The influence of process parameters on the velocity deviation difference (VDD) in profile extrusion: (**a**) billet temperature; (**b**) extrusion speed.

Figure 15a shows that, as the billet temperature increases, the VDD of the profile extrusion decreases, and the uniformity of the extrusion flow velocity improves; Figure 15b shows that, as the extrusion speed increases, the VDD decreases. Increasing the extrusion speed results in high-speed shear flow, dominating the deformation process; here, the temperature rise during extrusion compensates for the heat dissipation of the die, eliminating the lag in metal flow velocity at the edges and improving the uniformity of the extrusion flow velocity.

### 3.5. Optimization of Extrusion Process Parameters Based on Response Surface Methodology (RSM)

#### 3.5.1. Determination of Design Variables and Optimization Objectives

The billet temperature, extrusion speed, and billet length were selected as design variables for a three-factor, three-level Box–Behnken experimental design [35]. The extrusion load (which F represents), speed deviation (which VDD represents), average strain (which $\bar{\varepsilon }$ represents) of the profile cross-section, and temperature difference (which ΔT represents) of the profile cross-section were chosen as optimization objectives for multi-objective optimization. The synergistic effects of the four process parameters on the extrusion formability of the profile were investigated. The design variables and their ranges are shown in Table 5. Based on the optimal process window determined from the thermal processing diagram (Figure 10), the three levels of the billet temperature, *T*, were set as 360 °C, 390 °C, and 420 °C. Based on the significant influence of the extrusion speed, *v*, on the temperature of the extruded profile (Figure 12b), and to prevent a scenario where the overburning temperature was exceeded, three levels of *v* were designed: 0.1 mm/s, 0.3 mm/s, and 0.5 mm/s. Based on the capacity of the extrusion equipment, the three levels of the billet length, *L*, design were 350 mm, 575 mm, and 800 mm, respectively.

#### 3.5.2. Box–Behnken Experimental Design

This paper selects the Box–Behnken experimental design for response surface analysis. Based on the experimental design results, 15 process parameter modifications and numerical simulations were carried out, with Table 6 presenting the complete calculation results.

#### 3.5.3. Response Surface Model

The second-order response surface equation fitted by the least squares method is expressed as follows:

(15) $$y=\beta_{0}+\sum_{i=1}^{n}\beta_{i}x_{i}+\sum_{i=1}^{n}\beta_{ii}x_{i}^{2}+\sum \sum_{p<i}\beta_{pi}x_{p}x_{i}+\varepsilon$$

In this equation, $x_{i}$ is the design variable, ε is the residual error, and $\beta_{0}$, $\beta_{i}$, $\beta_{\mathrm{ii}}$, and $\beta_{pi}$ are all the undetermined coefficients.

Based on the data in Table 6 and using Equation (15) as the base expression, the response surface functions of the extrusion load, F, the deviation value, VDD, of the profile cross-sectional velocity, the average strain, $\bar{\varepsilon }$, of the profile cross-section, and the temperature difference, ΔT, of the profile cross-section with respect to each design variable are obtained, as shown in Equations (16)–(19):

(16) $$\begin{array}{l} F=223.66-0.79T+194.07v+0.0137L-0.29Tv+2.8*10^{-5}TL+0.0176vL+\\ 8.92*10^{-4}T^{2}-54.52v^{2}+2.38*10^{-6}L^{2} \end{array}$$

(17) SD=40.39−0.031T−20.33v+0.0033L

(18) $$\begin{array}{l} \bar{\varepsilon }=-6.03+0.067T+2.27v+0.0023L-4.2*10^{-5}Tv-5.3*10^{-6}TL-7.72*10^{-4}vL-\\ 8*10^{-5}T^{2}-2.63v^{2}-7.41*10^{-9}L^{2} \end{array}$$

(19) $$\Delta T=-45.69+0.14T-1.85v+0.08L+0.01Tv-2.16*10^{-4}TL+0.027vL$$

**Figure 16:**
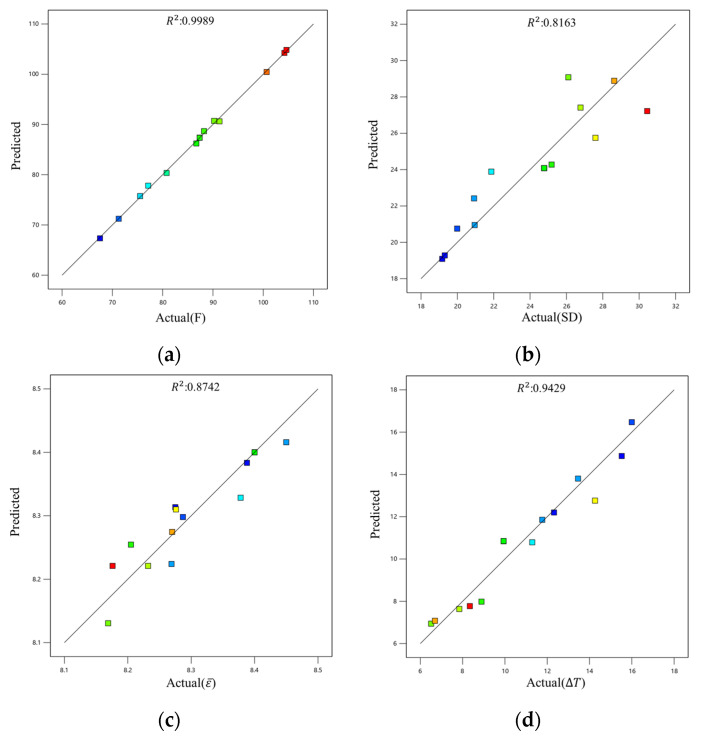
Comparison of actual values and predicted values of the four different response surfaces: (**a**) F; (**b**) VDD; (**c**) $\bar{\varepsilon }$; (**d**) Δ*T*.

The above four response surface models were used to predict, respectively, the four optimization objectives, F, VDD, $\bar{\varepsilon }$, and ΔT, under different extrusion process parameters. Figure 16 shows the comparison between the predicted values and actual values of the response surfaces for the various optimization objectives. Squares of different colors in the figure represent distinct actual data points.The results in Figure 16a,d indicate that the predicted values of the extrusion load, F, and the temperature difference, ΔT, of the profile cross-section are in good agreement with the actual values, suggesting that these response surface models are reliable. Figure 16b,c indicate, however, that the predicted values of the velocity deviation difference, VDD, and the average strain, $\bar{\varepsilon }$, of the profile cross-section deviate significantly from the actual values, necessitating a significance test evaluation.

The significance tests were conducted on the response surface models for key response indicators (extrusion load, flow velocity deviation, average strain, and cross-sectional temperature difference). The complete analysis tables are provided in Appendix A. The results indicate the following findings: The model for the extrusion load is highly statistically significant (F = 488.19, *p* < 0.0001, R^2^ = 0.9989). Among the factors, the extrusion speed (*v*) is the most influential factor affecting the extrusion load. The model for flow velocity deviation is statistically significant (F = 16.30, *p* = 0.0002). Its variation is primarily driven by extrusion speed (v). The model for average strain did not pass the significance test (*p* = 0.0754 > 0.05). This indicates that, within the range of process parameters studied, the billet temperature (*T*), extrusion speed (*v*), and ingot length (*L*) have no significant impact on the average strain across the profile cross-section. The model for cross-sectional temperature difference is statistically significant (F = 22.00, *p* < 0.0001). Extrusion speed (*v*) is again the most significant factor contributing to the cross-sectional temperature difference.

#### 3.5.4. Response Surface Analysis

**Figure 17:**
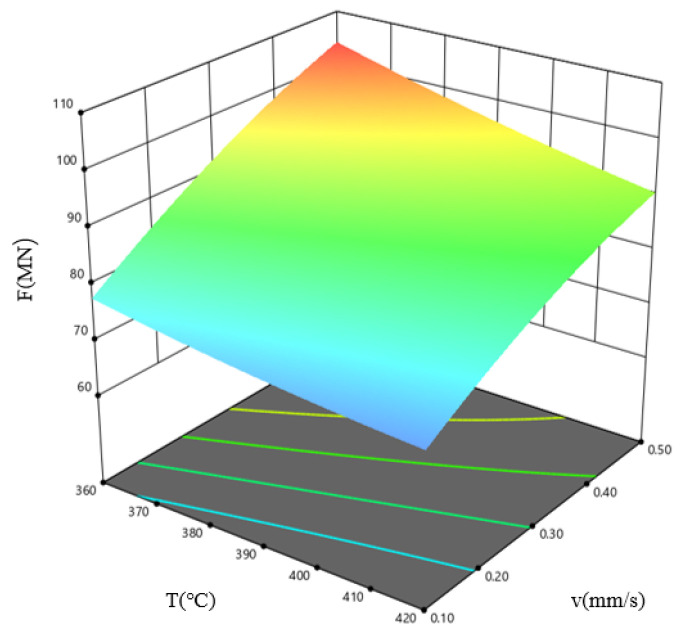
Response surface plot describing how *T* and *v* influence F, shown for the case where *L* = 575 mm.

Figure 17 shows the response surface plot for the combined influence of billet temperature, *T*, and extrusion speed, *v*, on the extrusion load, F, when the ingot length, *L*, is 575 mm. The results indicate that the extrusion load is minimal within the high-temperature, low-speed parameter window. As the billet temperature increases, the metal deformation resistance decreases, the plasticity increases, and the extrusion load decreases; at lower extrusion speeds, metal deformation occurs more slowly, internal stresses have time to adjust, the deformation heat effect is not significant, the temperature changes are smooth, and the metal deformation resistance is lower than at high speeds, resulting in reduced extrusion load.

**Figure 18:**
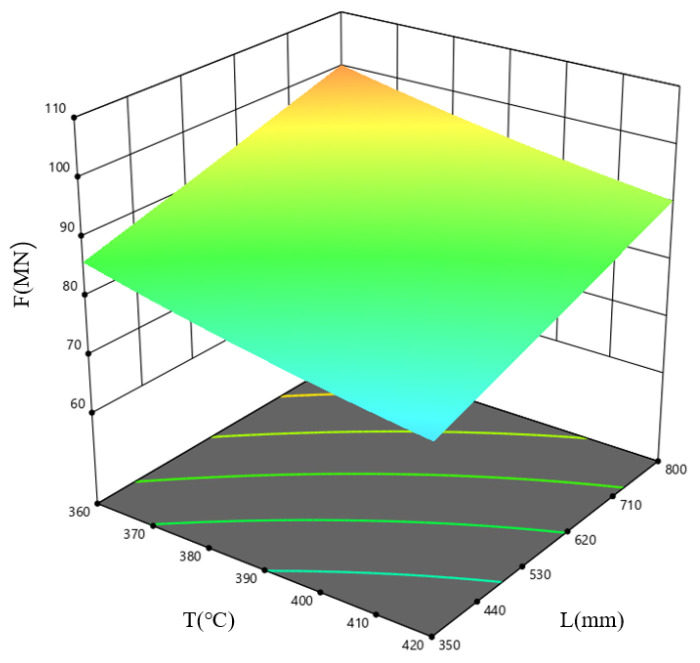
Response surface plot describing how *T* and *L* influence F, shown for the case where *v* = 0.3 mm/s.

Figure 18 shows the response surface plot for the combined influence of the billet temperature, *T*, and ingot length, *L*, on F at an extrusion speed, *v*, of 0.3 mm/s. The results indicate that the extrusion load is minimal within the high-temperature, short-ingot-length parameter window. As the ingot length increases, the contact area with the extrusion cylinder increases, the friction increases, the metal flow path lengthens, the flow resistance increases, and the pressure required to break through the extrusion increases.

**Figure 19:**
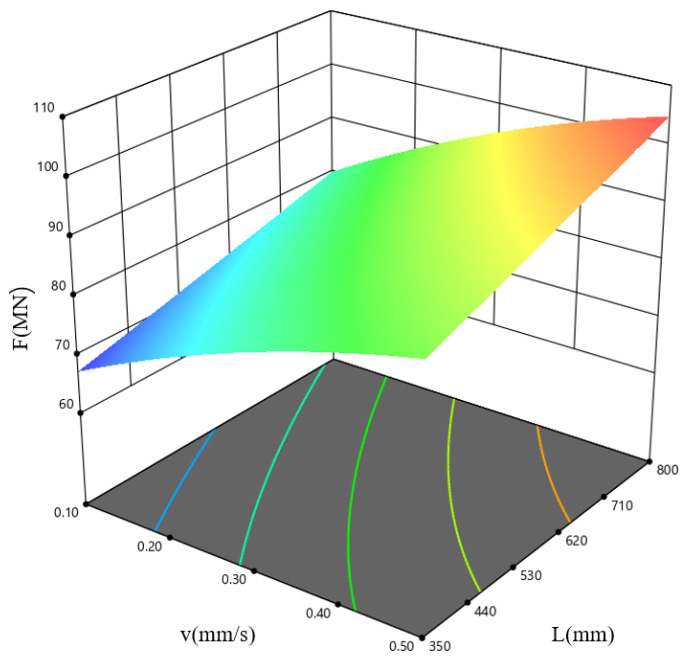
Response surface plot describing how *v* and *L* influence F, shown for the case where *T* = 390 °C.

Figure 19 shows the response surface plot for the combined influence of the extrusion speed, *v*, and the ingot length, *L*, on F at a billet temperature, *T*, equal to 390 °C. The results indicate that the extrusion load is minimal within the low-speed, short-ingot-length parameter window.

**Figure 20:**
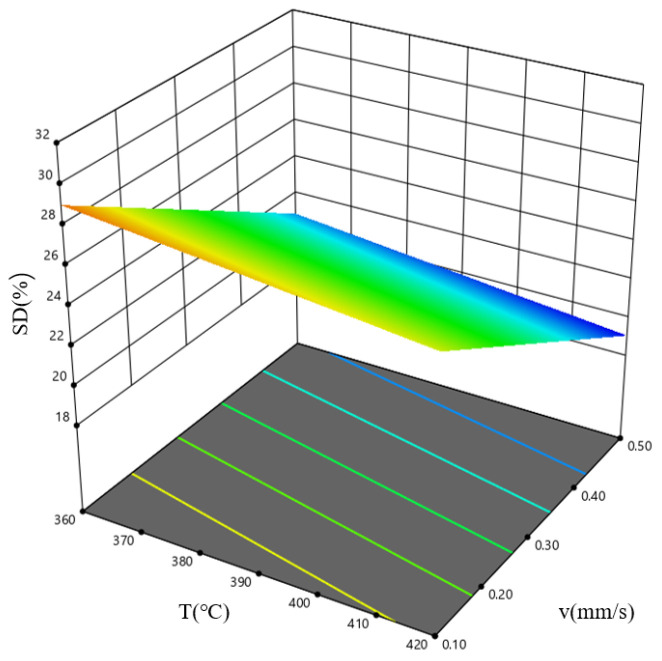
Response surface diagram describing how *T* and *v* influence the speed deviation difference, VDD, for the case where *L* = 575 mm.

Figure 20 shows the response surface plot for the combined influence of the billet temperature, *T*, and the extrusion speed, *v*, on the speed deviation difference, VDD, when the ingot length, *L*, is 575 mm. The results indicate that, within the high-temperature, high-speed parameter window, the speed deviation difference in the extruded profile cross-section is minimal, and the flow velocity is most uniform. As the billet temperature decreases, the metal deformation resistance increases, the plasticity decreases, the deformation becomes uneven, and the velocity deviation difference increases. As the extrusion speed decreases, the metal flow slows down, the flow time increases, and the accumulation of uneven deformation makes the uneven deformation more pronounced, resulting in an increase in the velocity deviation difference.

**Figure 21:**
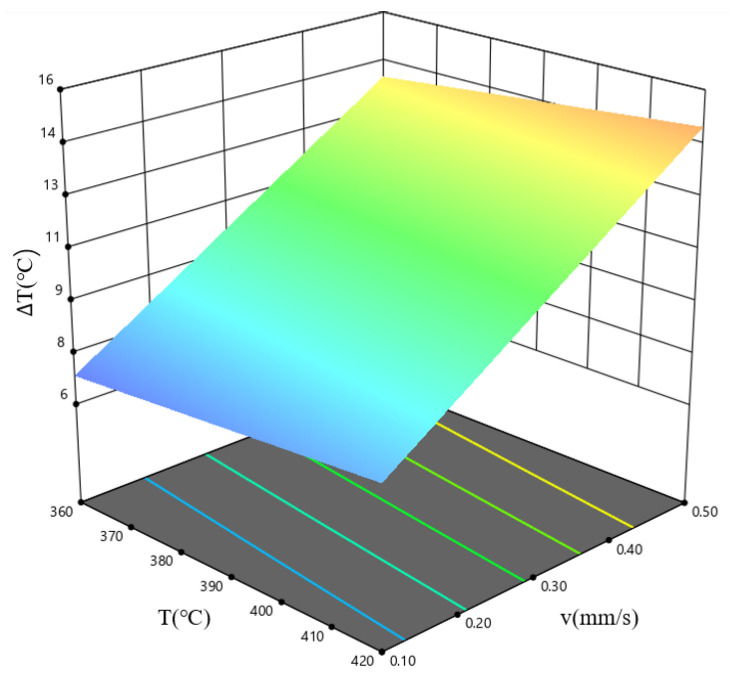
Response surface plots of the interaction between *T* and *v* on the profile temperature difference, for the case where *L* = 575 mm.

Figure 21 presents the response surface diagram for the combined influence of the billet temperature, *T*, and the extrusion speed, *v*, on the temperature difference, ΔT, of the extruded profile, when the ingot length, *L*, is 575 mm. The results indicate that the temperature difference across the extruded profile cross-section is minimal within the high-temperature, low-speed parameter window. As the billet temperature increases, the metal deformation resistance decreases, resulting in less deformation-induced heat generation and a smaller temperature difference across the extruded profile cross-section. Conversely, as the extrusion speed decreases, heat conduction becomes more uniform, leading to more balanced heat distribution and a smaller temperature difference across the extruded profile’s cross-section.

**Figure 22:**
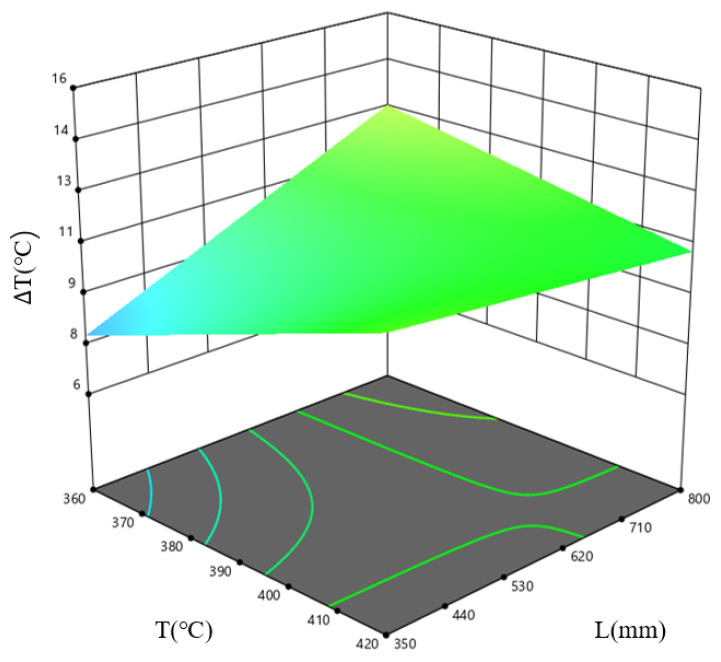
Response surface plots of the interaction between *T* and *L* on the profile temperature difference, for the case where *v* = 0.3 mm/s.

Figure 22 shows the response surface plot for the combined influence of the billet temperature, *T*, and ingot length, *L*, on ΔT at an extrusion speed, *v*, of 0.3 mm/s. The results indicate that this temperature difference is minimal within the low-temperature, short-ingot-length parameter window. This is because, as ingot length increases, the distance for heat conduction increases, leading to uneven heat distribution and a larger temperature difference across the profile cross-section; the lower the billet temperature, the lower the heat conduction capacity, resulting in less deformation-induced heat generation and a larger temperature gradient. This leads to the billet length having a more significant influence on the temperature difference across the profile cross-section.

**Figure 23:**
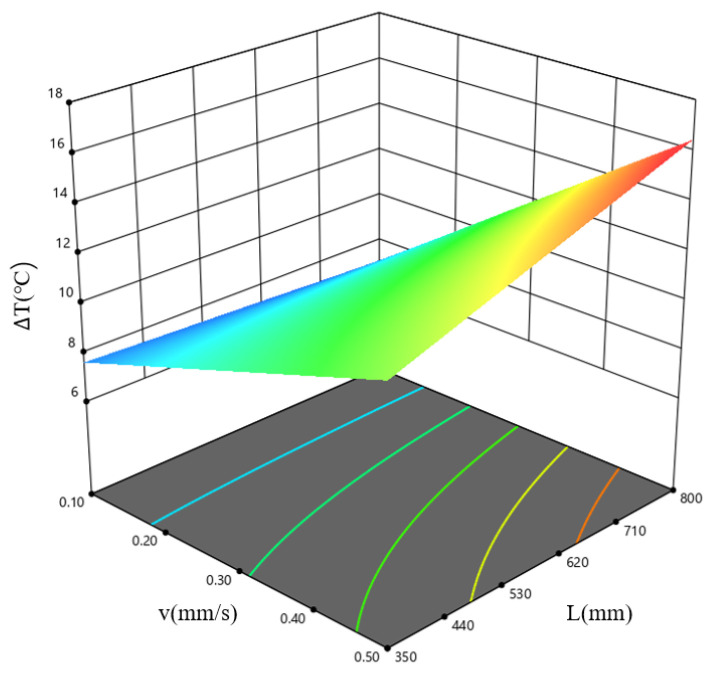
Response surface plots of the interaction between *v* and *L* on the profile temperature difference, for the case of 390 °C.

Figure 23 shows the response surface diagram for the combined influence of the extrusion speed, *v*, and the ingot length, *L*, on ΔT when the billet temperature, *T*, is 390 °C. The results indicate that the temperature difference in the cross-section of the extruded profile is minimal within the low-speed, short-ingot-length parameter window, consistent with the previous conclusion.

**Figure 24:**
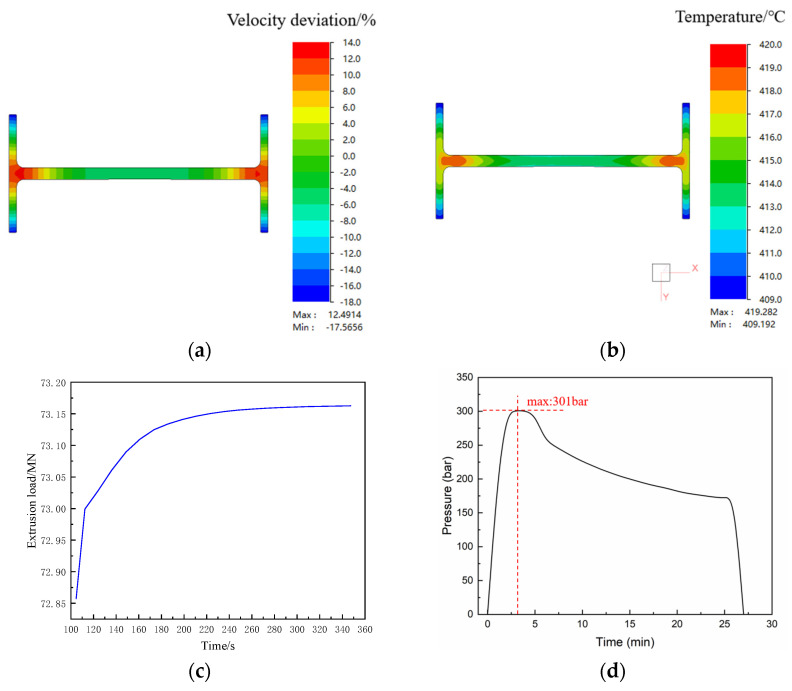
Prediction of the formability of extruded profiles under optimized extrusion process parameters: (**a**) speed deviation cloud chart; (**b**) extruded profile cross-section temperature field cloud map; (**c**) simulated extrusion load; (**d**) actual extrusion load.

Through multi-objective optimization of the extrusion load for aluminum alloy profiles using response surface methodology (RSM), the optimal parameters were determined: a billet temperature of 400 °C, an extrusion speed of 0.20 mm/s, and an ingot length of 350 mm. Under these conditions, the extrusion load, the profile velocity deviation difference (VDD), and the cross-sectional temperature difference in the extruded profile all reached relatively minimum values. The predicted extrusion load at this point is 73.29 MN, with a VDD of 24.96% and a cross-sectional temperature difference of 9.48 °C. Figure 24 presents the simulation results of the optimized extrusion process parameters based on the RSM model. The optimized parameters yield a VDD of 29.88%, an extrusion load of 73.16 MN, and a cross-sectional temperature difference of 10.06 °C. Notably, the error between the simulated and predicted extrusion load is less than 1%, and the error for the cross-sectional temperature difference is less than 6%, further confirming the significance and reliability of the response surface model. Based on the aforementioned optimization results, a practical extrusion test of the profile was carried out. The extrusion equipment used was an 80 MN extrusion press, with a rated working oil pressure of 320 bar. Figure 24d shows that the actual breakthrough pressure during extrusion was 301 bar. After a proportional calculation, the actual breakthrough extrusion load was 75.25 MN, while the simulated prediction was 73.29 MN. The prediction error is less than 5%, indicating that the finite element simulation results are reliable.

The use of optimized process parameters significantly reduces the extrusion load, which facilitates the engineering application of high-magnesium low-density aluminum alloy profiles. However, the error between the simulated and predicted VDD is less than 17%, indicating a relatively large discrepancy. In the model, only the extrusion speed is identified as a significant term affecting VDD, while other parameters show no significance. This suggests that improving the uniformity of the extrusion flow velocity requires optimization of the die structure, as the extrusion process parameters have limited influence on the uniformity of extrusion flow velocity.

## 4. Conclusions

(1) The flow stress of the Al–10Mg–3Zn alloy increases with increasing strain rate and decreases with increasing deformation temperature, indicating that the alloy exhibits positive strain rate sensitivity. A temperature-corrected true stress–strain curve was used to establish the Arrhenius strain compensation constitutive model for the Al–10Mg–3Zn alloy, which helps us to more accurately describe the rheological behavior of this alloy at elevated temperatures.
(2) The thermal processing diagrams constructed for different strain values can accurately predict the safe processing temperature range and strain rate range for the homogeneous Al–10Mg–3Zn alloy; moreover, the working temperature range of 360–420 °C and the strain rate range of 0.004–0.123 s^−1^ were determined to be optimal for this alloy.
(3) The extrusion speed has a greater influence on the extrusion temperature of the profile and the uniformity of the cross-sectional temperature than the billet temperature. As the ingot length, extrusion speed, and friction coefficient increase, the extrusion load shows a linear upward trend. As the billet temperature increases, the extrusion load shows a linear downward trend. Increasing the billet temperature and extrusion speed can improve the uniformity of the extrusion flow rate of the profile.
(4) The extrusion process parameters optimized by the response surface method were set at a billet temperature of 400 °C, an extrusion speed of 0.20 mm/s, and a billet length of 350 mm. Under these conditions, the predicted extrusion breakthrough load is 73.29 MN, which deviates by less than 5% from the experimentally measured value. This optimized parameter set effectively lowers the required extrusion load, leading to a significant increase in the trial molding success rate and thereby facilitating the engineering application of high-magnesium, low-density aluminum alloy profiles.

## Figures and Tables

**Table 1:** Measured chemical composition of the high-magnesium low-density aluminum alloy examined in the present work.

Elements	Mg	Zn	Mn	Cr	Zr	Ti	Fe	Si	Al
Content	9.7	2.9	0.41	0.09	0.14	0.1	≤0.01	≤0.01	Balance

**Table 2:** Combinations of extrusion temperature, extrusion speed, and overall friction coefficient examined in the present work.

Combination Number	I	II	III	IV	V	VI	VII
Extrusion speed/mm/s	0.3	0.3	0.3	0.1	0.5	0.3	0.3
Billet temperature/°C	360	390	420	390	390	390	390
Friction factor	1	1	1	1	1	0.5	1.5
Ingot length/mm	800	800	800	800	800	800	800

**Table 3:** Combinations of billet length and friction coefficient of the extrusion cylinder examined in the present work.

Combination Number	A	B	C	D	E
Extrusion speed/mm/s	0.3	0.3	0.3	0.3	0.3
Billet temperature/°C	390	390	390	390	390
Ingot length/mm	800	350	550	800	800
Coefficient of friction of extrusion cylinder	1	1	1	0.1	0.5

**Table 4:** Al–10Mg–3Zn alloy polynomial fitting parameters.

α		Q		n		A	
$\alpha_{0}$	0.0108	$Q_{0}$	160,458	$n_{0}$	5.74	$A_{0}$	3.99 × 10^11^
$\alpha_{1}$	−0.0073	$Q_{1}$	−75,667	$n_{1}$	−18.96	$A_{1}$	−3.69 × 10^12^
$\alpha_{2}$	0.0200	$Q_{2}$	−7381	$n_{2}$	76.1	$A_{2}$	1.4 × 10^13^
$\alpha_{3}$	−0.0141	$Q_{3}$	737,554	$n_{3}$	−160.36	$A_{3}$	−2.49 × 10^13^
$\alpha_{4}$	−0.0022	$Q_{4}$	−1,291,052	$n_{4}$	167.69	$A_{4}$	2.36 × 10^13^
$\alpha_{5}$	0.0048	$Q_{5}$	642,526	$n_{5}$	−67.91	$A_{5}$	−9.7 × 10^12^

**Table 5:** Design variables and value ranges for the three-factor, three-level Box–Behnken experimental design.

Design Variable	Value Ranges
Billet temperature *T* (°C)	360–420
Extrusion speed *v* (mm/s)	0.1–0.5
Ingot length *L* (mm)	350–800

**Table 6:** Calculation results as obtained for the Box–Behnken experimental design. Columns 2–4 show the independent variables of experimental input, and columns 5–8 show the dependent variables of simulation output.

Experiment Number	T (°C)	*ν* (mm/s)	*L* (mm)	F (MN)	VDD (%)	$\bar{\varepsilon }$	ΔT (°C)
1	360	0.1	575	77.139	26.1029	8.169	6.511
2	420	0.1	575	71.2617	30.4403	8.176	8.341
3	360	0.5	575	104.213	20.95196	8.269	13.457
4	420	0.5	575	91.31	19.16665	8.275	15.525
5	360	0.3	350	86.6928	25.183	8.205	8.891
6	420	0.3	350	75.5111	20.92162	8.45	11.765
7	360	0.3	800	100.682	27.5956	8.276	14.262
8	420	0.3	800	90.2429	21.8673	8.378	11.291
9	390	0.1	350	67.5451	26.7742	8.232	7.843
10	390	0.5	350	88.2312	19.30918	8.388	12.322
11	390	0.1	800	80.7862	28.6261	8.27	6.691
12	390	0.5	800	104.633	19.98956	8.287	15.999
13	390	0.3	575	87.3589	24.773	8.4	9.938
14	390	0.3	575	87.3589	24.773	8.4	9.938
15	390	0.3	575	87.3589	24.773	8.4	9.938

## Data Availability

The original contributions presented in this study are included in the article. Further inquiries can be directed to the corresponding authors.

## Appendix A. Complete Analysis of Significance Tests

### Appendix A.1. Test Framework

In the significance testing of the response surface models, a model is considered statistically significant and well-fitted when it has a sufficiently large F-statistic, a *p*-value less than 0.05, a coefficient of determination (R^2^) and an adjusted coefficient of determination that are close to 1, and a signal-to-noise ratio that exceeds 4.

### Appendix A.2. Extrusion Load Response Model

**Table A1:** Analysis of variance (ANOVA) for the extrusion load response model.

Factor	Sum of Squares	Degree of Freedom	Mean Square	F-Statistic	*p*-Value
Model	1716.12	9	190.68	488.19	<0.0001
*T*	204.03	1	204.03	522.38	<0.0001
*v*	1050.08	1	1050.08	2688.50	<0.0001
*L*	425.79	1	425.79	1090.15	<0.0001
*Tv*	12.34	1	12.34	31.59	0.0025
*TL*	0.1379	1	0.1379	0.353	0.5783
*vL*	2.50	1	2.50	6.39	0.0526
*T* ^2^	2.38	1	2.38	6.09	0.0567
*v* ^2^	17.56	1	17.56	44.95	0.0011
*L* ^2^	0.0537	1	0.0537	0.1375	0.7259
Residual	1.95	5	0.3906		
Correction	1718.07	14			

Correlation coefficient R^2^ = 0.9989, correction factor R^2^ = 0.9968, signal-to-noise ratio = 73.4980.

Analysis: The model is highly significant. The extrusion speed (*v*) has the largest obtained F-value (2688.50), i.e., the most pronounced effect recorded on the extrusion load. The interaction term, *Tv*, and the quadratic term, *v*^2^, are also significant, while other higher-order terms are not significant.

### Appendix A.3. Profile Cross-Sectional Velocity Deviation Response Model

**Table A2:** Analysis of variance (ANOVA) for the velocity deviation response model.

Factor	Sum of Squares	Degree of Freedom	Mean Square	F-Statistic	*p*-Value
Model	143.50	3	47.83	16.30	0.0002
*T*	6.91	1	6.91	2.36	0.1530
*v*	132.24	1	132.24	45.06	<0.0001
*L*	4.34	1	4.34	1.48	0.2495
Residual	32.28	11	2.93		
Correction	175.78	14			

Correlation coefficient R^2^ = 0.8163, correction factor R^2^ = 0.7663, signal-to-noise ratio = 11.2938.

Analysis: The model is significant but explains only 81.63% of the variance. Extrusion speed (*v*) is the significant controlling factor for velocity deviation (*p* < 0.0001), while billet temperature and length have no significant effect.

### Appendix A.4. Profile Cross-Sectional Average Strain Response Model

**Table A3:** Analysis of variance (ANOVA) for the average strain response model.

Factor	Sum of Squares	Degree of Freedom	Mean Square	F-Statistic	*p*-Value
Model	0.1009	9	0.0112	3.86	0.0754
*T*	0.0162	1	0.0162	5.58	0.0645
*v*	0.0173	1	0.0173	5.96	0.0585
*L*	0.0005	1	0.0005	0.1764	0.6919
*Tv*	2.5 × 10^−7^	1	2.5 × 10^−7^	0.0001	0.9930
*TL*	0.0051	1	0.0051	1.76	0.2418
*vL*	0.0048	1	0.0048	1.66	0.2534
*T* ^2^	0.0193	1	0.0193	6.66	0.0493
*v* ^2^	0.0410	1	0.0410	14.13	0.0132
*L* ^2^	5.192 × 10^−7^	1	5.192 × 10^−7^	0.0002	0.9898
Residual	0.0145	5	0.0029		
Correction	0.1154	14			

Correlation coefficient R^2^ = 0.8742, correction factor R^2^ = 0.6479, signal-to-noise ratio = 6.4910.

Analysis: The model *p*-value is 0.0754 (>0.05), which fails the significance test. None of the investigated process parameters (*T*, *v*, *L*) or their interactions or quadratic terms show stable or significant predictive powers for the average cross-sectional strain.

### Appendix A.5. Profile Cross-Sectional Temperature Difference Response Model

**Table A4:** Analysis of variance (ANOVA) for the temperature difference response model.

Factor	Sum of Squares	Degree of Freedom	Mean Square	F-Statistic	*p*-Value
Model	120.50	6	20.08	22.00	0.0001
*T*	1.81	1	1.81	1.98	0.1972
*v*	97.42	1	97.42	106.73	<0.0001
*L*	6.89	1	6.89	7.54	0.0252
*Tv*	0.0142	1	0.0142	0.0155	0.9039
*TL*	8.54	1	8.54	9.36	0.0156
*vL*	5.83	1	5.83	6.39	0.0354
Residual	7.30	8	0.9128		
Correction	127.80	14			

Correlation coefficient R^2^ = 0.9429, correction factor R^2^ = 0.9, signal-to-noise ratio = 14.6017.

Analysis: The model is significant. The extrusion speed (*v*) is the most critical factor, inducing the cross-sectional temperature difference (F = 106.73). The interaction terms, *TL* and *vL*, also show some significance, but their effects are far smaller than the main effect, the extrusion speed.

## References

[1] Deng Y., Zhang X., Li J., Wang H., Liu Z., Chen L., Yang S., Zhou F. (2019). Development of aluminum and aluminum alloy. Chin. J. Nonferrous Met..

[2] Fang H., Liu H., Sun J., Wang R., Xu Y., Zhao L., Cheng X., Li M. (2023). Research Status and Development Trend of 5xxx Series Aluminum Alloys. Mater. Rep..

[3] Wang Z.T. (2015). Handbook of Aluminum Materials for Aerospace Vehicles.

[4] Qi Z., Wu R., Wang G., Qiang W., Legan H. (2016). Application of aluminum alloys in shipbuilding and marine engineering. Light Alloy Fabr. Technol..

[5] Zhu Z.J. (2018). Overview of new aluminum-magnesium and aluminum-zinc-magnesium alloy materials for aerospace applications. China Strateg. Emerg. Ind..

[6] Yang S.J., Dai S.L. (2005). Review and prospect of the development of aviation aluminum alloys. Mater. Rev..

[7] Alil A., Popović M., Radetić T., Zrilić M., Romhanji E. (2015). Influence of annealing temperature on the baking response and corrosion properties of an Al–4.6 wt% Mg alloy with 0.54 wt% Cu. J. Alloys Compd..

[8] Zhang D., Zhang Z., Pan Y., Jiang Y., Zhuang L., Zhang J., Zhang X. (2020). Current-driving intergranular corrosion performance regeneration below the precipitates solvus temperature in Al–Mg alloy. J. Mater. Sci. Technol..

[9] Guo C., Zhang H., Li S., Chen R., Nan Y., Li L., Wang P., Li B., Cui J., Nagaumi H. (2021). Evolution of microstructure, mechanical properties and corrosion behavior of Al-4Mg-2Zn-0.3Ag (wt.%) alloy processed by T6 or thermomechanical treatment. Corros. Sci..

[10] Meng C.Y. (2016). Corrosion Resistance Mechanism and Sheet Development of Zn-Containing 5xxx Aluminum Alloy for Ship Application.

[11] Meng C., Zhang D., Cui H., Zhuang L., Zhang J. (2014). Mechanical properties, intergranular corrosion behavior and microstructure of Zn modified Al–Mg alloys. J. Alloys Compd..

[12] Meng C.Y., Zhang D., Liu P.P., Zhuang L.Z., Zhang J.S. (2018). Microstructure characterization in a sensitized Al-Mg-Mn-Zn alloy. Rare Met..

[13] Carroll M., Gouma P., Mills M., Daehn G., Dunbar B. (2000). Effects of Zn additions on the grain boundary precipitation and corrosion of Al-5083. Scr. Mater..

[14] Zhao J.-W., Luo B.-H., He K.-J., Bai Z.-H., Li B., Chen W. (2016). Effects of minor Zn content on microstructure and corrosion properties of Al−Mg alloy. J. Central South Univ..

[15] Meng C., Zhang D., Zhuang L., Zhang J. (2016). Correlations between stress corrosion cracking, grain boundary precipitates and Zn content of Al–Mg–Zn alloys. J. Alloys Compd..

[16] Khomutov M.G., Pozdniakov A.V., Churyumov A.Y., Barkov R.Y., Solonin A.N., Glavatskikh M.V. (2021). Flow stress modelling and 3D processing maps of Al4.5Zn4.5Mg1Cu0.12Zr alloy with different scandium contents. Appl. Sci..

[17] Lei C., Wang Q., Ebrahimi M., Li D., Tang H., Zhang N., Cai H. (2022). Hot Deformation Behavior and Processing Maps of an As-Cast Al-5Mg-3Zn-1Cu (wt%) Alloy. Materials.

[18] Yang Y., Niu G., Zhao P., Wang G., Zou L. (2025). Influence of hot deformation rate on microstructure of Al-Mg-Zn alloy. Light Alloy Fabr. Technol..

[19] Xiao Y.-H., Guo C., Guo X.-Y. (2011). Constitutive modeling of hot deformation behavior of H62 brass. Mater. Sci. Eng. A.

[20] Wu H., Wen S.P., Huang H., Wu X.L., Gao K.Y., Wang W., Nie Z.R. (2016). Hot deformation behavior and constitutive equation of a new type Al-Zn-Mg-Er-Zr alloy during isothermal compression. Mater. Sci. Eng. A.

[21] Zhang C., Zhang L., Shen W., Liu C., Xia Y., Li R. (2016). Study on constitutive modeling and processing maps for hot deformation of medium carbon Cr–Ni–Mo alloyed steel. Mater. Des..

[22] Zhou L., Zhang P., Wang Q., Xiao B., Ma Z., Yu T. (2019). Multi-Scale Study on the Fracture Behavior of Hot Compression B4C/6061Al Composite. Acta Metall. Sin..

[23] Xiang S., Liu D.-Y., Zhu R.-H., Li J.-F., Chen Y.-L., Zhang X.-H. (2015). Hot deformation behavior and microstructure evolution of 1460 Al–Li alloy. Trans. Nonferrous Met. Soc. China.

[24] Chen G., Chen L., Zhao G., Zhang C., Cui W. (2017). Microstructure analysis of an Al-Zn-Mg alloy during porthole die extrusion based on modeling of constitutive equation and dynamic recrystallization. J. Alloys Compd..

[25] Lin Y.C., Xia Y.C., Chen X.M., Chen M.S. (2010). Constitutive descriptions for hot compressed 2124-T851 aluminum alloy over a wide range of temperature and strain rate. Comput. Mater. Sci..

[26] Peng N.Q., Tang G.B., Liu Z.D. (2012). Correcting method of flow stress curve for hot compression. Hot Work. Technol..

[27] Zhao D. (1993). Temperature correction in compression tests. J. Mater. Process. Technol..

[28] Wang Y., Zhao G., Xu X., Chen X., Zhang C. (2019). Constitutive modeling, processing map establishment and microstructure analysis of spray deposited Al-Cu-Li alloy 2195. J. Alloys Compd..

[29] Yang H., Bu H., Li M., Lu X. (2021). Prediction of Flow Stress of Annealed 7075 Al Alloy in Hot Deformation Using Strain-Compensated Arrhenius and Neural Network Models. Materials.

[30] Wang S.-Q., Zhao X., Ren X.-W., Zhang Z.-M., Tian X.-D., He Y.-Y. (2023). Hot Deformation Behavior and Processing Map Considering Strengthening Effect for Al–10.0Zn–3.0Mg–2.8Cu Alloy. Materials.

[31] Wu M., Wei W., Zuo R., Wen S., Shi W., Zhou X., Wu X., Gao K., Huang H., Nie Z. (2023). Effect of Zr and Er Addition on the Microstructural Evolution of a Novel Al—Mg—Zn—Er—Zr Alloy during Hot Compression. Materials.

[32] Jeong H., Kim H., Kim W. (2021). Processing maps (with flow instability criterion based on power-law breakdown) integrated into finite element simulations for evaluating the hot workability of 7075 aluminum alloy. Mater. Today Commun..

[33] Lakshmi A.A., Rao C.S., Gangadhar J., Srinivasu C., Singh S.K. (2017). Review of Processing Maps and Development of Qualitative Processing Maps. Mater. Today Proc..

[34] Yang Q., Liu W., Zhang Z., Huang G., Liu X. (2018). Hot Deformation Behavior and Processing Maps of AA7085 Aluminum Alloy. Rare Met. Mater. Eng..

[35] Liu H., Liu T., Chen Z., Wang Y., Zhang J., Li X., Zhou W., Yang F., Xu S., Sun L. (2022). Optimization of Extrusion Process for Large-Sized Thin-Walled Aluminum Profiles with Ribs Based on Experimental Design and Response Surface Methodology. Forg. Stamp. Technol..

